# Establishing an integrated prevention and control system to ensure zero nosocomial infections – an analysis of the logistics support in controlling nosocomial infections during the hospital’s fight against the COVID-19 epidemic

**DOI:** 10.3205/dgkh000422

**Published:** 2022-10-17

**Authors:** Shi Zhilin, Peng Xin, Huang Min, Li Mingjian, Chen Jiongliang, Lai Guozhi, Xiong Xiaohong, He Qing, Liu Lei

**Affiliations:** 1 Third People’s Hospital of Shenzhen, Southern University of Science and Technology, Shenzhen City, China

**Keywords:** novel coronavirus, COVID-19, hospital logistics, nosocomial infection control, public events, supporting role

## Abstract

**Aim:**

To analyze the role of the logistics support services in nosocomial infection control during emergency periods, with a focus on job responsibilities including the organization of vehicle parking, supply of hospital meals, washing of medical bedding and clothing, disposal and management of medical sewage and waste, elevator services, disinfection of air conditioning systems, disinfection and cleaning of ambulances, management of hospital buildings, storage of sterilization supplies, reception and delivery of oxygen cylinders and protection of staff health as examples.

**Methods:**

The adjustment and optimization of the emergency support system and working mode as part of hospitals’ response to major public emergencies were summarized, and the vital supporting role of the logistics support services in nosocomial infection control was analyzed.

**Results:**

The logistics support services played a crucial role in ensuring the high-performance operations of the hospitals and control of nosocomial infections, resulting in the excellent outcome of “zero infection” among hospital staff.

**Conclusion:**

Establishing a safe, flexible and efficient system for the logistics support services is important in ensuring an effective response by hospitals to health emergencies.

## Zusammenfassung

**Zielsetzung:**

Es geht um die Analyse der unterstützenden Rolle von Logistikprozessen zur Bekämpfung nosokomialer Infektionen in Notfallsituationen, z. B. in Bezug auf die Organisation des Parkens von Fahrzeugen, die Bereitstellung von Krankenhausmahlzeiten, das Waschen medizinischer Bettwäsche und Kleidung, die Entsorgung medizinischer Abwässer und Abfälle, den Fahrstuhldienst, die Desinfektion von Klimaanlagen, die Desinfektion und Reinigung von Krankenwagen, das Management von Krankenhausgebäuden, die Lagerung von Sterilgut, die Entgegennahme und Lieferung von Sauerstoffflaschen und den Personalschutz.

**Methode:**

Die Anpassung und Perfektionierung des Notfallsystems als Teil der Reaktion des Krankenhauses auf große öffentliche Notfälle wurden zusammengefasst und die wichtige Rolle von Logistikprozessen bei der Bekämpfung nosokomialer Infektionen analysiert.

**Ergebnisse:**

Logistikprozesse spielten eine entscheidende Rolle bei der Sicherstellung des Hochleistungsbetriebs des Krankenhauses und zur Prävention nosokomialer Infektionen, was zu dem ausgezeichneten Ergebnis der „Null-Infektion“ beim Krankenhauspersonal führte.

**Schlussfolgerung:**

Die Einrichtung eines sicheren, flexiblen und effizienten Logistiksystems ist wichtig, um die effektive Reaktion des Krankenhauses auf medizinische Notfälle zu gewährleisten.

## Introduction

As the only designated hospital in the city to admit and treat patients diagnosed with COVID-19, the Third People’s Hospital of Shenzhen (the National Clinical Medical Center for Infectious Disease and the Second Affiliated Hospital of South University of Science and Technology) has been treating all COVID-19 patients in Shenzhen. As of March 22, 2020, the number of confirmed cases reached 439, of which 411 had been discharged from the hospital after recovery. While the hospital was exerting their best efforts to control the pandemic, the logistics department immediately initiated emergency support measures and played an important role in ensuring the intensive operation of the hospital and the control of nosocomial infections. These measures greatly contributed to the notable results of “zero nosocomial infections”. The purpose of this paper is to analyze the logistic efforts in supporting the control of nosocomial infections during the hospital’s fight against the COVID-19 pandemic by summarizing the establishment of an integrated supporting system against public emergencies by the logistics department of the hospital. 

## Method

Appropriate scientific methods were used in terms of washing, collection and delivery of medical bedding and clothing, staff meal ordering and delivery, sewage disposal, elevator service, use of air conditioners and ventilators, collection and transportation of medical waste, ambulance decontamination, access and parking of the vehicles, security and safety inspection, safety specifications for the disinfection products, department security management, collection and delivery of oxygen cylinders, and health protection of the migrant workers. 

## Results

Obviously, the logistics support services played a crucial role in ensuring the high-performance operations of the hospitals and control of nosocomial infections, resulting in the excellent outcome of “zero infection” among the hospital staff.

### 1. Establishment of a logistics support service system for emergency responses in the hospital 

Shortly after the hospital formally decided to fight against the COVID-19 pandemic, the logistics department took the following actions: launched the Public Health Emergency Response Plan, improved the organizational structure, established an emergency response team for COVID-19 and created a clear emergency feedback system. Moreover, the *Guideline for Logistics Support to COVID-19 Emergency of the Third People’s Hospital of Shenzhen* was compiled by summarizing the key links of the supporting services along with the relevant national policies, laws and regulations as well as the actual admissions and treatment status of the hospital. This guideline comprises over 10 items related to the logistics support to control nosocomial infections, including the processes of washing, collecting and delivering bedding and clothing, meal ordering and delivery plan, sewage disposal plan, elevator service plan, requirements for the use of air conditioners and ventilators, collection and transportation of medical waste, ambulance decontamination process, access and parking program of the vehicles at the hospital, security and safety inspection plan, safety specifications for disinfection products, department security management plan, collection and delivery processes of oxygen cylinders, and health protection and management plan of the migrant workers, which may be adjusted or updated from time to time. The implementation of this guideline is aimed at preventing nosocomial infections and providing robust logistics support services.

### 2. Main practices and experiences

#### 2.1 Access and parking program of the vehicles at the hospital

**Figure 1:**
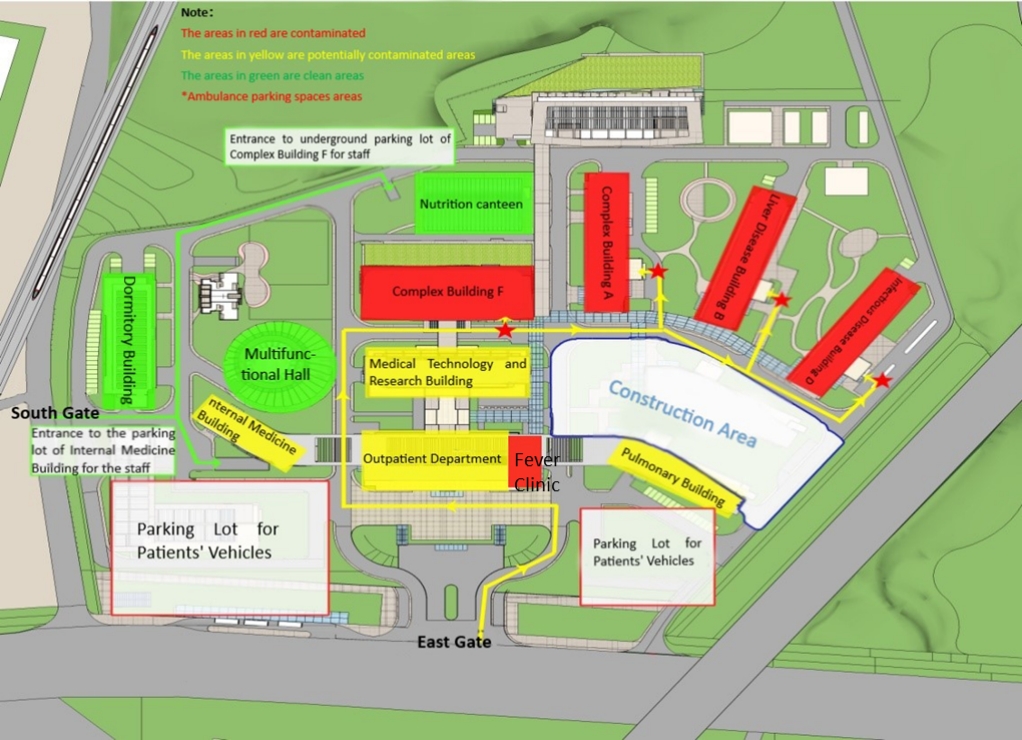
Plan view of the vehicle direction and parking lots

Since it is necessary to separate the vehicles of the hospital staff from those of the patients, the vehicle management mode at the hospital was adjusted such that the vehicles of the hospital staff entered and exited the hospital from the South Gate and those of the patients from the East Gate. Besides, the underground parking garage was allocated to the staff, while the ground-level parking lot was allocated to the patients. In addition, fixed ambulance parking spaces were designated around the in-patient buildings (Figure 1 (Fig. 1)).

The access and parking program of the vehicles at the hospital was formulated as part of prevention and control during the COVID-19 pandemic. Meanwhile, the entry and exit routes as well as the parking areas for the transport vehicles for medical bedding and clothing were defined.

#### 2.2 Adjustment of the processes of washing, collecting and delivering medical bedding and clothing

**Figure 2:**
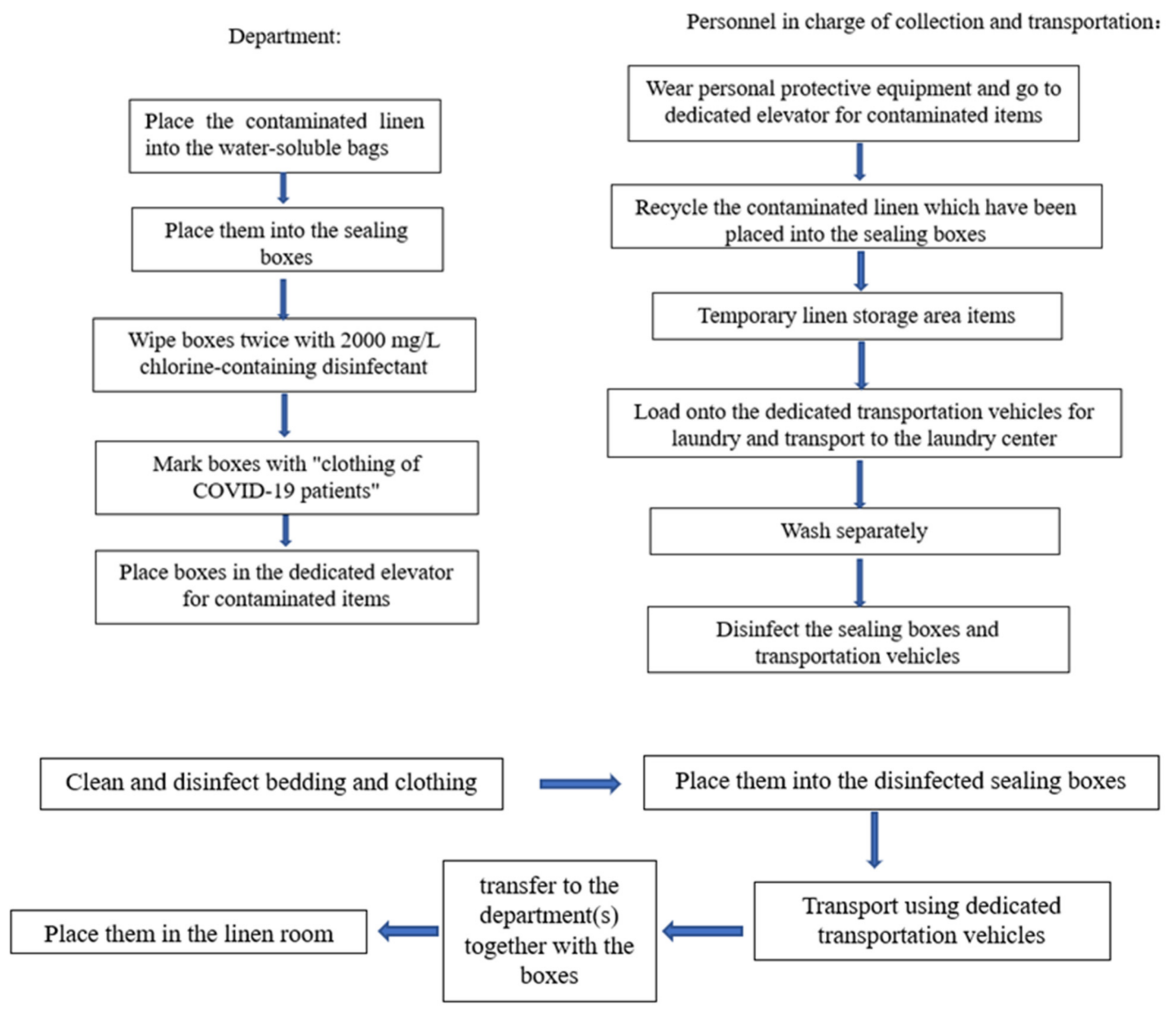
Washing, collection and delivery processes of bedding and clothing from departments related to admission and treatment of COVID-19 patients. (1) Recycling of contaminated bedding and clothing; (2) Transportation of the bedding and clothing back to the hospital

The guideline for nosocomial infections included distinguishing the COVID-19-related departments from conventional departments, and introducing the laundry, collection and delivery processes of clothing as part of the prevention and control measurements during the COVID-19 pandemic. Moreover, a detailed working plan in response to the prevention and control of the pandemic was developed based on timely communication with the outsourced laundry company. These adjustments entailed the follows: Instead of the original storage area, an elevator was designated as the storage place for contaminated bedding and clothing items, collected without counting, from the departments related to the admission and treatment of COVID-19 patients. Specifically, all these items were put into water-soluble bags and then into sealed boxes, whose outer surfaces were wiped twice with 2,000 mg/L chlorinated disinfectant solution before being transferred. After contacting the contaminated bedding and clothing, the sealed boxes were soaked in the 2,000 mg/L chlorinated disinfectant for over 30 minutes. If the boxes were found to have blood stains, they would be wiped with 10,000 mg/L chlorinated disinfectant, then soaked and disinfected in the 2,000 mg/L chlorinated disinfectant for more than 30 minutes (Figure 2 (Fig. 2)).

To protect the staff responsible for recycling bedding and clothing from being exposed to risk and to avoid the potential nosocomial infections during the prevention and control of the COVID-19 pandemic, 93 sealed boxes (130 L) were employed as storage containers for the water-soluble bags with contaminated bedding and clothing. This facilitated the prevention of potential damage to the water-soluble bags due to various reasons, such as friction during transport, and to minimize the probability of pollutant exposure.

**Figure 3:**
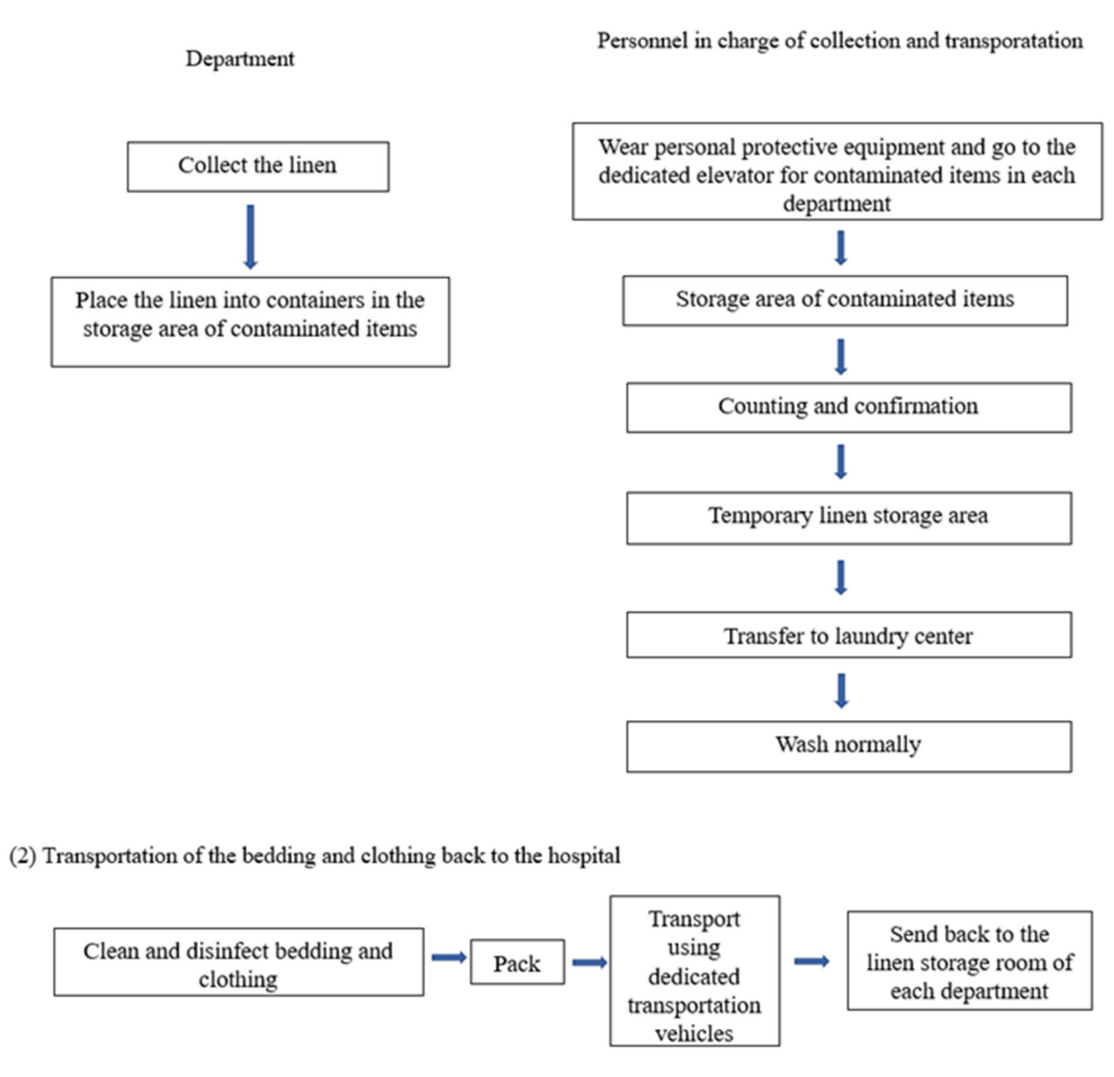
Washing, collection and delivery processes of bedding and clothing from conventional departments. (1) Recycling of contaminated bedding and clothing; (2) Transportation of the bedding and clothing back to the hospital

The staff responsible for bedding and clothing collection and delivery were trained in the prevention of nosocomial infections. They were instructed to correctly wear personal protective equipment and appropriately execute hand hygiene in a timely manner (Figure 3 (Fig. 3)).

Training in the processes of washing, collecting and delivering bedding and clothing was carried out. The transportation of bedding and clothing was performed according to the training, and transport vehicles were disinfected as per the relevant regulations. The operation procedures were revised, requiring the nursing units to help in disinfecting and transferring the sealed boxes to the dedicated elevator for contaminated items, in order to keep the staff responsible for bedding and clothing collection and delivery from entering the endemic area.

**Figure 4:**
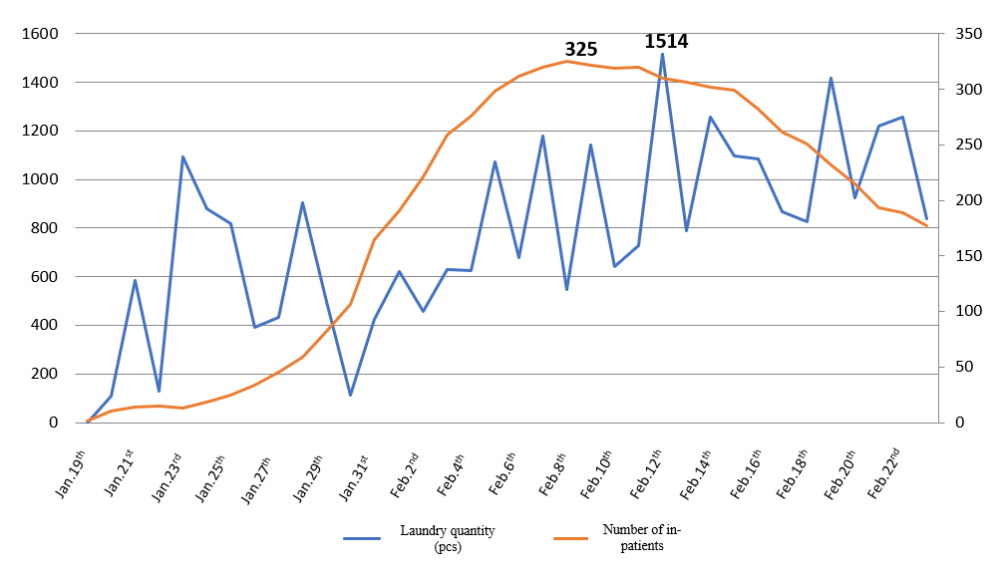
Laundry workload of COVID-19 related departments of the General Services Section.

Figure 4 (Fig. 4) gives an overview of the number of laundry pieces accrued and the number of treated patients. 

#### 2.3 Meal ordering and delivery

A food delivery system was adopted for the medical staff working in the COVID-19-related departments. The food for the patients was delivered to the Nurses’ Station and transferred to the recipients by the nurses of each department. To avoid possible cross-infection, educational campaigns were conducted to inform the in-patients that it was not allowed to bring in food from outside the hospital. Optional meals were canceled, and a simple meal was introduced instead. It was made compulsory for people to wear face masks when entering the staff canteen.

Staff members were recommended to choose take-out food and eat in the respective departments. If they ate the food in the canteen, only one person was allowed at each table, similar to the seating arrangement of the college entrance examinations.

The purchase of supporting insulation devices was coordinated, the flow of food preparation was properly simplified, and the delivery time was shortened.

**Figure 5:**
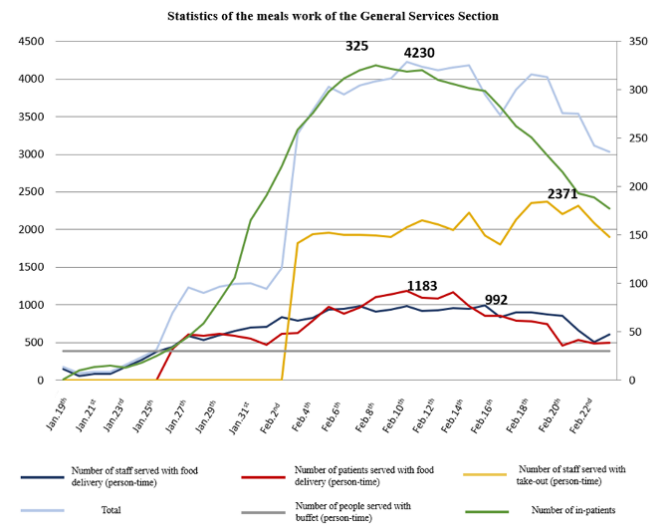
Breakdown of meals served by the General Services Section

Figure 5 (Fig. 5) gives an overview of the patients and staff involved in the food supply.

#### 2.4 Medical sewage disposal

The administrator of the medical sewage treatment station was responsible for the daily addition of sodium hypochlorite to the sterilizing pools in the pretreatment area of each building and at the station, as well for the purpose of sterilization and disinfection, such that the amount of residual chlorine was 8 mg/L at the main drain outlet.

An online monitoring system was used to record and monitor the amount of residual chlorine and pH value of the raw water in the regulating pool as well as the COD value, pH value, total amount of residual chlorine, number of fecal coliforms per liter of water, and daily discharge amount to ensure there were no abnormalities and that the discharge standards were met.

The administrator of the medical sewage treatment station carried out a daily COVID-19 nucleic acid test on the samples from the raw water entering the regulating pool at the medical sewage treatment station and the treated water from the station to ensure absence of the COVID-19 virus (i.e., negative nucleic-acid test results) in the medical sewage from the hospital and that the discharge standards were met.

Table 1 (Tab. 1) summarizes the usage of chlorinated disinfectants before and during the control of the outbreak.

#### 2.5 Medical waste management

**Figure 6:**
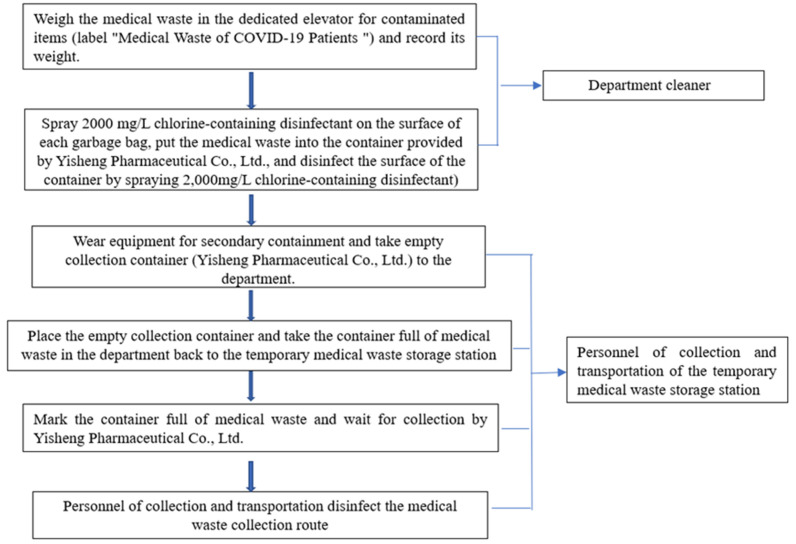
Flow chart of the intra-hospital transportation of medical waste

A “Four Designated” rule was applied to the management of medical waste, which can be explained as follows: Medical waste was collected and transported by designated personnel, transferred though a designated passage, disinfected by designated personnel and transported by designated vehicles. To reduce the risk of a nosocomial cross-infection, the whole process was strictly disinfected: medical waste was collected by the logistics personnel of the departments that handle COVID-19 patients. They collected, sealed, weighed, labeled (marked as "medical waste from COVID-19 patients") and spray-disinfected each bag of waste from these departments (the surface of each bag of high-risk medical waste was sprayed with 2,000 mg/L chlorinated disinfectant). The medical waste was then placed into the containers that were subsequently sealed. Later, the surface of the collection container was disinfected by spraying with the same disinfectant (Figure 6 (Fig. 6)).

Medical waste was transported from the departments to the temporary medical waste storage station by the personnel responsible for medical waste collection and transportation, who entered the temporary medical waste storage room of the departments via the fixed route to deliver and transport the collection containers. Next, the personnel labeled the container surface of the high-risk medical waste from the departments with the tag “Dedicated Container for Highly Infectious Waste” in red in the temporary storage station, followed by another round of disinfection. After completing these steps, the personnel returned to the collection and transportation route and conducted spray disinfection.

The collection and transportation personnel from the professional medical waste disposal company arranged dedicated vehicles to collect and transfer the medical waste from the hospital.

**Figure 7:**
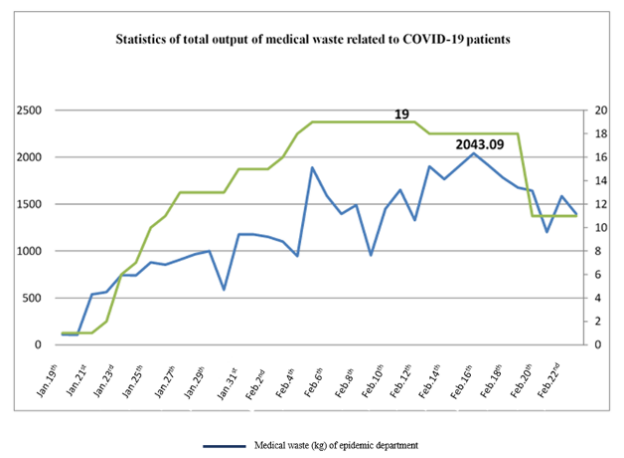
Total output of medical waste related to COVID-19 patients

Following the admission of COVID-19 patients to the hospital, the total amount of medical waste at the hospital has significantly increased (Figure 7 (Fig. 7)). From January 19 to February 23, the total amount of disposed medical waste was 31,548.04 kg. 

#### 2.6 Elevator service plan

Different elevators were selected for exclusive use by the medical staff, normal patients, COVID-19 patients or operations. Additional signs and guides were added, and all the elevators were disinfected regularly. Further, the elevators were remotely controlled using contactless methods such as mobile phones.

#### 2.7 Air conditioning and exhaust system

The medical staff area and the ward area were equipped with independent fresh-air systems. An independent system was set up for the air-conditioner drains, and the coil system of the air conditioning in each room was made independent as well. The fresh-air system was installed with manual and electric air valves with BA system, such that each room could be independently and remotely controlled. The air conditioning filter was regularly cleaned and disinfected.

An electric air-valve system and exhaust fan were installed and interconnected in the exhaust duct of each ward’s bathroom, and the valve was automatically closed after the shutdown of the electric air valve. A high-efficiency sterilizing exhaust fan was installed at the exhaust outlet on the roof of the building to facilitate discharge after disinfection.

#### 2.8 Ambulance decontamination process

**Figure 8:**
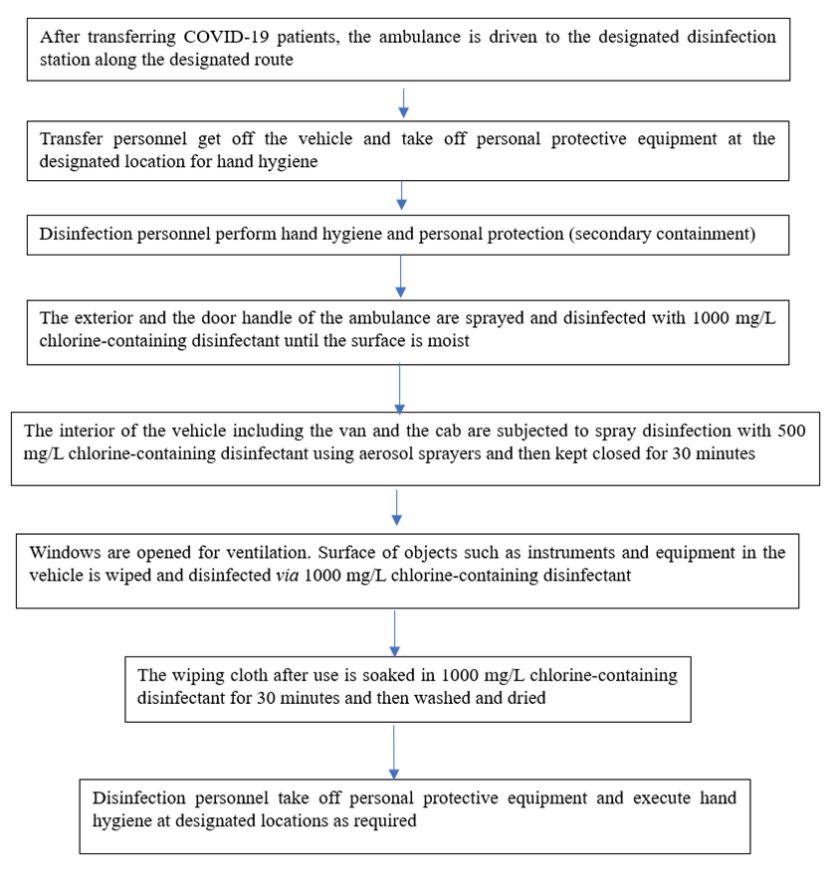
Flow chart of cleaning and disinfection of ambulances after use

After transferring COVID-19 patients, the ambulance was driven to the designated disinfection station along the allocated route. After exiting the vehicle, the transfer personnel removed their personal protective equipment at the designated location to perform hand hygiene. The disinfection personnel performed hand hygiene and personal protection (secondary containment) as well. Later, the exterior and door handles of the ambulance were disinfected with 1,000 mg/L chlorinated disinfectant spray until the surface was moist. Moreover, the interior of the vehicle, including the van and the cab, was sprayed with 500 mg/L chlorinated disinfectant using aerosol sprayers and then kept shut for 30 minutes. Subsequently, the windows were opened for ventilation, and the surfaces of the objects, such as the instruments and equipment in the vehicle, were wiped and disinfected using 1,000 mg/L chlorinated disinfectant and then wiped with clean water after 30 minutes. Following this, the wiping cloth was soaked in 1,000 mg/L chlorinated disinfectant for 30 minutes and then washed and dried. Finally, the disinfection personnel removed their personal protective equipment and executed hand hygiene at the designated locations. Figure 8 (Fig. 8) gives an overview of the process of cleaning and disinfection of ambulances. 

#### 2.9 The building visit and accompanying management and safety inspection system

A policy of not accompanying the patients was implement-ed in the in-patient area for COVID-19 patients, and no personnel (except for the medical staff and specially approved personnel) were allowed to enter and exit the admission and treatment area of the COVID-19 patients. Round-the-clock security posts were set up in the patient elevator hall in a timely manner to guarantee safe operation of the endemic area. COVID-19 patients were not allowed to leave the department and would be stopped in time if they tried to go outside. It was mandatory that the security staff in the departments and buildings related to the admission and treatment of COVID-19 patients be familiar with the telephone numbers of the head nurses and nurse stations of the corresponding departments. If the in-patients went out for medical examinations, the security personnel would help to evacuate irrelevant personnel in the area.

While random and unannounced safety inspections used to be carried out at any time before the pandemic, the safety inspection of the whole hospital was carried out twice a day during the special period of the fight against COVID-19. Overall inspections covered the safety guarantee, security personnel on duty, traffic organization, material storage and other situations in the key areas of the hospital. Such areas mainly included entrances and exits, fire monitoring centers, ground-level and underground parking lots, outpatient and emergency treatment areas, attending gate sentry of each in-patient building, room for water, electricity and special equipment, oxygen station and others.

#### 2.10 Safety specifications for disinfectants

In order to effectively inactivate the virus during the critical period of the fight against the COVID-19 pandemic, 75% ethanol, chlorinated disinfectants, peracetic acid and other disinfection products were widely used in various departments. Disinfectants pose safety hazards, as they are flammable, toxic, corrosive, and can easily lead to accidents, such as fire, poisoning and burns if stored or used improperly. Therefore, it was necessary to promptly issue safety specifications for the use of these products. The procurement department strictly checked the product label, specification, letter of commitment for the product quality and safety and other documents and completed the warehousing procedures. Each department was responsible for the storage, use and emergency disposal of the disinfectants in their own areas, and the received amount, use and consumption of the products were routinely updated in detail. The logistics management department conducted special supervision and inspection of all the disinfectants at the hospital, recorded any problems that may have been found, provided feedback to the relevant department as soon as possible, and urged rectification on time to prevent the occurrence of disinfectant-related accidents.

#### 2.11 Specifications for the collection and delivery of oxygen cylinders

In order to reduce the infection risks of the paramedical staff, the collection and delivery processes of medical oxygen cylinders were adapted. Specifically, 2 L, 4 L and 10 L medical oxygen cylinders were delivered by the security staff to the nurses’ station of each department, and the staff from each department brought them to the isolation wards and took out the empty ones. After disinfection, the cylinders were delivered to and returned by the security personnel. Regarding the 40 L oxygen cylinder, it was delivered by the department staff to the isolation ward. If it needed to be sent by the security staff to the ward, then corresponding protective equipment would be provided by the department. The department staff would check whether the security personnel wore their protective equipment correctly and accordingly allow them to enter.

#### 2.12 Health protection and management plan for migrant workers in general affairs and logistics when receiving COVID-19 patients

To standardize the health protection behaviors of migrant workers in general affairs and logistics during their work in our hospital and to avoid cross-infection, a health protection and management plan was formulated for them. New migrant workers had to declare in advance, fill out the health screening form and undergo a physical examination. After that, only those with qualified physical examination results were hired.

During their work in our hospital, the personnel of the outsourcing company were required to correctly wear the COVID-19 protective equipment in strict accordance with the relevant guidelines for personal protective equipment. Employee behavior was controlled when they were outside the hospital, and they were not allowed to organize or attend parties. If several people were stationed in our hospital for short-term work, they were recommended to rent dormitories around the hospital to avoid using public transportation for a long time. Moreover, their body temperature was measured, and their health conditions were reported every day before the commencement of work. Any discovered abnormalities would be immediately put under centralized management by the specialized departments. Non-resident personnel were not allowed to enter the hospital. If they had to enter designated areas at the hospital due to special circumstances, their health conditions and place of birth were inquired in advance, which would be submitted to and approved by the centralized management department to allow their entry.

## Conclusions

The importance of nosocomial infection management is increasingly prominent in the operation and management of modern hospitals regarding the improvement of medical care quality, ensuring patient safety and maintaining the health interests of medical staff [1]. Controlling nosocomial infections during the fight against the COVID-19 pandemic is more important because it is associated with the treatment of diagnosed patients, the examination and screening of suspected patients, and the self-protection of the medical and paramedical staff. Many measures and work requirements proposed by the nosocomial infection department need to be implemented and completed by the logistics department, and this determines the efficacy and quality of the nosocomial infection control to a large extent.

As the primary transmission route of COVID-19 is through person-to-person contact and through direct contact with respiratory droplets from infected patients, limiting crowds to avoid cross-infection is one of the effective measurements to control the pandemic. Since its outbreak, our hospital established and reorganized various specifications and procedures of logistics work in advance under unified requirements of nosocomial infection control. Scientifically sound adjustments were made in work areas including washing, collection and delivery of medical bedding and clothing, staff meal ordering and delivery, sewage disposal, elevator service, use of air conditioners and ventilators, collection and transportation of the medical waste, ambulance decontamination, access and parking of vehicles, security and safety inspection, safety specifications for the disinfectants, department security management, collection and delivery process of oxygen cylinders, and health protection of migrant workers. While the hospital was working to ensure the orderly, standard diagnosis and treatment of the patients, the initial panic of the staff towards the pandemic was also effectively eased, and the mentality was changed from “I am afraid” to “I am not afraid”. Thus, both patients and employees felt that the hospital was a safe place.

During the fight against the pandemic, the logistics department of the Third People's Hospital of Shenzhen started the emergency guarantee and service in time and formed a logistics emergency management mechanism of “unified command, immediate response, orderly coordination and efficient operation”. The logistics department paid close attention to the changes in the pandemic situation at all times and adjusted the emergency plan accordingly. This kind of response helped the department to avoid any loopholes in the control of nosocomial infections, which reflects a strong emergency coordination capacity. Therefore, excellent results of “zero infection” in the hospital were achieved, accumulating valuable experience for handling public health emergencies by medical care institutions in the future [2], [3].

## Notes

### Competing interests

The authors declare that they have no competing interests.

### Acknowledgements

The authors would like to thank the departments of the Third People’s Hospital of Shenzhen (including the medical department, nursing department and nosocomial infection department) for their support and the medical workers of the hospital for their persistent hard work.

## Figures and Tables

**Table 1:**
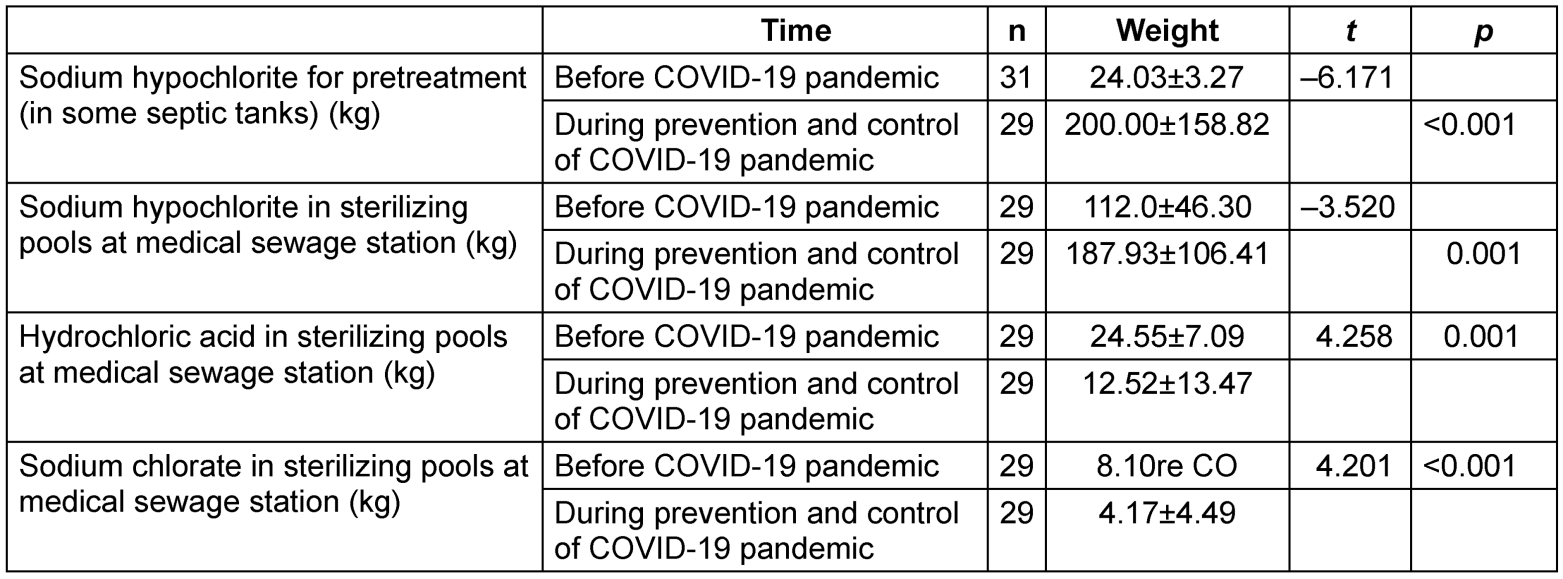
Comparison of the usage of chlorinated disinfectants in various regions before the outbreak and during the prevention and control of the outbreak
